# Resilience to Global Health Challenges Through Nutritional Gut Microbiome Modulation

**DOI:** 10.3390/nu17030396

**Published:** 2025-01-22

**Authors:** Erika Isolauri, Kirsi Laitinen

**Affiliations:** 1 Department of Clinical Medicine, Faculty of Medicine, University of Turku, 20520 Turku, Finland; eriiso@utu.fi; 2 Department of Paediatrics and Adolescent Medicine, Turku University Hospital, Kiinamyllynkatu 4-8, 20520 Turku, Finland; 3 Nutrition and Food Research Center & Institute of Biomedicine, Faculty of Medicine, University of Turku, 20520 Turku, Finland

**Keywords:** nutrition, pregnancy, infant, gut microbiota, probiotics

## Abstract

As the world faces an escalating challenge of non-communicable diseases (NCDs), with phenotypes ranging from allergic chronic immuno-inflammatory diseases to neuropsychiatric disorders, it becomes evident that their seeds are sown during the early stages of life. Furthermore, within only a few decades, human obesity has reached epidemic proportions and now represents the most serious public health challenge of our time. Recent demonstrations that a growing number of these conditions are linked to aberrant gut microbiota composition and function have evoked active scientific interest in host-microbe crosstalk, characterizing and modulating the gut microbiota in at-risk circumstances. These efforts appear particularly justified during the most critical period of developmental plasticity when the child’s immune, metabolic, and microbiological constitutions lend themselves to long-term adjustment. Pregnancy and early infancy epitomize an ideal developmental juncture for preventive measures aiming to reduce the risk of NCDs; by promoting the health of pregnant and lactating women today, the health of the next generation(s) may be successfully improved. The perfect tools for this initiative derive from the earliest and most massive source of environmental exposures, namely the microbiome and nutrition, due to their fundamental interactions in the function of the host immune and metabolic maturation.

## 1. Introduction: Two Hypotheses Setting the Scene

Recent epidemiological, experimental, and clinical studies have directed the scientific interest toward nutritional modulation of the gut microenvironment at critical stages of the child’s maturation. The interventions in question focus on the developmental period of the first 1000 days, from pregnancy through to infancy. These efforts are based on the concept that early-life programming may permanently modify the structure and function of the regulatory systems of the body and contribute to adult disease, according to The Developmental Origins of Health and Disease hypothesis [1], also known as The Barker hypothesis [2]. Indeed, the NCDs tend to manifest in adulthood and have their origins in childhood. More recent data, however, witness the rise in the prevalence of NCDs, particularly allergic sensitization, chronic inflammatory diseases, and overweight development, that are already present in childhood and adolescence [3].

The World Health Organization (WHO) defined obesity as an epidemic in 1997 and set an international target for halting the rise in obesity rates by 2025; a target clearly missed [4,5]. The surge of obesity embodies the critical period of programming: obesity is frequently detected among women of reproductive age. More than half of pregnant women are overweight or obese. Maternal obesity is associated with high birth weight and increased risk of childhood obesity, while obesity in children is likely to persist into adulthood [6,7,8].

Lately, it has become apparent that the NCDs share common environmental risk factors and immunological features that frequently coexist. All these conditions have been linked with a distinct gut microbiota composition that differs from that of healthy individuals [9]. As a result, The Disappearing Microbiota hypothesis can rationalize the emergence of NCDs by pointing to the depletion of microbial contact in the modern industrialized world, resulting from changes in hygiene, antibiotic use, diet, lifestyle, and living conditions [3,10].

The aim of this narrative review is to present scientific evidence on contributing factors and opportunities for microbiota modulation during critical early life periods with subsequent potential health benefits.

## 2. Key Determinants of Child Maturation Toward Health: The Gut Barrier and the Healthy Microbiota

The gastrointestinal tract strongly contributes to the total immunologic capacity of the host, while the gut microbiota exercises a function in controlling and maintaining intestinal homeostasis (reviewed in: [11]). First, the mucosal surface of the gastrointestinal tract forms an important organ of host defense. The establishment of the gut microbiota, particularly at delivery and during breastfeeding, contributes to the primary maturation of these functions [11,12]. The dilemma of the immature and immunologically inexperienced newborn child is to acquire a dual function: a delicate balance of effective alertness to pathogens while concomitantly creating a disease-free coexistence with the indigenous microbiota.

Second, in addition to its main function, both via the digestion and absorption of nutrients to meet the metabolic requirements and the demands of normal growth and development, the gut barrier provides a bidirectional crossing point between the internal environment and the constant challenge from the antigens of the outside environment. The healthy maturation of a balanced barrier requires, however, these external stimuli, particularly through the establishment of the microbiome and early nutrition [11,12,13].

**Figure 1:**
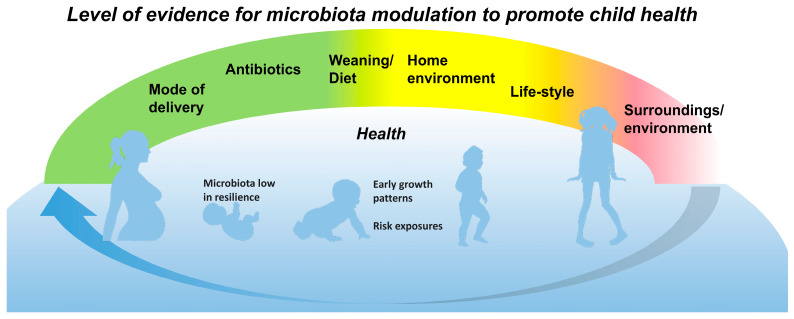
The period of the first 1000 days, from pregnancy throughout infancy, offers a promising time window for interventions aiming to improve the long-term health of the child. The current evidence from clinical trials demonstrates a wide range of potential targets for microbiota modulation during this continuum (green color), while that of exposures and nutritional interventions beyond this period has been limited (red color). The blue arrow points to the intergenerational aspect of microbiome development and the risk of non-communicable diseases.

The model of early nutrition is the healthy full-term vaginally-delivered breast-fed child. Conversely, the risk circumstances and the key intervention targets (Figure 1) may be identified in the model of a modern neonate, who is exposed to the unfavorable nutritional environment during pregnancy, born preterm or by cesarean section or devoid of important immunomodulatory compounds of breast milk, and who thereby may lack an age-appropriate and environment-adjusted microbe contact [14]. This may substantially increase the child’s risk of developing NCDs. The clinical manifestations may include allergy, asthma, obesity, type 1 diabetes, and neuropsychiatric disorders [15,16].

## 3. The Journey Toward Health: The First 1000 Days

As the critical time window to exert a programming effect on later health falls around birth [1,2,17], pregnancy and early infancy represent an optimal target for interventions aiming to reduce the risk of NCDs in the child (Figure 1). Early life exposures and circumstances that are known to perturb the gut microbiota have been consistently linked with lasting alterations in the immune and metabolic phenotype as well as neural pathways (reviewed in: [11]).

### 3.1. Pregnancy

Preliminary research data show how the mother’s health and weight gain during pregnancy, along with the mode of delivery, impact a child’s health, with the microbiome as one key mechanism [11]. It was demonstrated that the pregnancy-associated proinflammatory factors, attributable to increased weight accumulation especially in the third trimester, also affect the gut microbiota composition and activity [18]. However, also, the reverse might equally be true, i.e., the microbiota changes may act as the driving force inducing metabolic alterations during pregnancy. Actually, in obese adult individuals, a distinctive deviated gut microbiota composition prevails, with adjustments following weight gain or weight loss [19]. Too much weight gain during pregnancy has been shown to amplify the microbiota deviation [20]. These features appear beneficial during pregnancy in shunting metabolic fuels to fetal growth, while the same profiles epitomize metabolic syndrome in a non-pregnant situation. It is of note, however, that aberrant gut microbiota may also relate to the onset of gestational diabetes mellitus [21], although not all studies demonstrate such deviation [22], a condition that may seriously affect the health of both mother and child even in years to come [23].

Considering environmental exposures, it is of note that our environmental biodiversity is declining at an alarming rate, and so is our contact with environmental biodiversity, due to antibiotics and changes in hygiene and diet [3,24]. Recent research suggests that the gut microbiota of pregnant women has also changed over time [25]. The study in question documents that the women pregnant in 1997 exhibited significantly higher microbiota richness and diversity as compared to those pregnant in 2007 and 2017. The study provides direct evidence for a decline in gut microbiota diversity over two decades in the same geographical area and population. Thus, the study tends to provide a parallel for the decline in biodiversity within our natural surroundings that coincides with the evolution of the allergy and obesity epidemics, thereby corroborating The Disappearing Microbiota hypothesis [1,3,10,24,26].

### 3.2. Delivery Mode and Antibiotic Exposure

The mother imprints the child’s microbiota development at delivery and during breastfeeding [27]. In neonates delivered by cesarean section, the initial colonizers originate from the hospital environment and oral or skin microbiota of the mother. Cesarean section-delivered subjects tend to exhibit gut microbiota perturbations characterized by decreased diversity and delayed colonization as well as altered immune maturation throughout infancy [11,14]. This may translate into an increased risk of NCD; increased occurrences of obesity, asthma, and inflammatory bowel disease have been reported in children born by cesarean section [28,29]. The clinical significance of these phenomena is obvious, in view of the high frequency of this delivery mode in many parts of the world, while this worldwide trend seems to continue.

Delivery by cesarean section is frequently accompanied by antibiotic contact with the neonate. Antibiotics frequently represent a child’s first detrimental exposure, affecting nearly half of neonates [30,31]. The concern of antibiotic exposure peaks in early infancy: maternal antibiotic consumption during pregnancy and delivery, via transmission of antibiotic resistance genes and gut microbiota, shapes the neonatal gut [32]. As a result, antibiotic exposure at delivery may initiate the development of proinflammatory microbiota and the unfavorable immunological and metabolic maturation of the child. Neonatal antibiotic exposure is associated with significant differences in the gut microbiota, particularly in decreased abundance and diversity of fecal Bifidobacteria. A low abundance of Bifidobacteria in early infancy, again, was shown to precede the development of allergy and the overweight state or obesity [15,16].

Epidemiological studies in children suggest an age-dependent association between frequent antibiotic use and the development of being overweight or obese [33,34,35]. The heightened NCD risk presented by the cesarean section delivery may arise from or become consolidated by deviant breast milk composition: distinct profiles have been documented between mothers delivering vaginally compared to those undergoing cesarean section delivery [20]. Moreover, the breast milk microbiota composition differs between the types of cesarean delivery, elective vs. non-elective cesarean section. This observation points to an essential regulatory role of the physiological labor process, stress, and/or hormonal signals on a balanced microbiota composition. In addition to antibiotics, medication during delivery such as pain or anesthetic medication may contribute to breast milk composition and the child’s primary microbiota [36].

### 3.3. Breastfeeding

Bifidobacteria typify the gut microbiota of the healthy breast-fed infant. It has become evident that *Lactobacillaceae* and *Bifidobacteriaceae* decrease upon the introduction of solid foods and the transition to family foods [37], with gut microbial diversity and richness significantly increasing concomitantly. It appears that *Bifidobacterium* colonization frequencies and counts among mother–infant pairs show a relationship [38]. Above pregnancy and birth, the maternal microbiota guides the compositional development of the child’s microbiota via breastfeeding, covering the critical period of immunological and metabolic maturation. Bogaert and colleagues [27] studied the microbial transmission from mother to child and showed that a significant proportion of infants’ microbiota can be attributed to any of the maternal source communities. Reduced transfer of fecal microbes in children delivered by cesarean section was compensated for by other niches, with a main role for the breast milk microbiota.

The individual structure of the mother’s milk of lipids and protein components is a reflection of the immediate environment and diet. These components interact and examining their exclusive effects may prove impossible. Early environment and exposures by the enteral route, particularly early diet, induce adaptive modifications in the microbiota composition and activity. Microbiota changes, again, control energy acquisition and storage and may contribute to the gut immunological milieu. As a unique active compound, breast milk is the first source of nondigestible oligosaccharides, which are known as human milk oligosaccharides (HMOs). HMOs carry a direct effect on the gut microbiota of the child by modulating immune responses, acting as substrates for specific bacteria, and interfering with pathogen-, virus-, and toxin-binding receptors (reviewed in: [9]). Particularly for the growth of *Bifidobacterium* and *Bacteroides,* HMOs are of fundamental importance.

Thus far, we do not know whether the changes in the microbiome underlie the pathogenesis of NCDs or are results thereof confronting the question of causality.

The proof of causality requires experimental documentation of the mechanisms and clinical intervention studies in humans in different populations with rigorous and detailed documentation of the environment the infant is exposed to, the major determinant of which being early nutrition.

## 4. Microbiota Modulation Through Diet During Pregnancy and Breastfeeding

### 4.1. General Notions on Diet—Gut Microbiota Interactions

A healthy balanced diet meets the needs for growth and development in children. Research interest in pediatric nutrition is also directed toward the potential to reduce the risk of diseases. Observational studies have indicated a potential that infant/child gut microbiota modulation may be a relevant means to promote child normal growth, development, and health and thus lower the risk for NCD.

The dietary components that have been studied include particular fats (mainly long-chain polyunsaturated fatty acids, LC-PUFA, e.g., in the format of fish oil), probiotics, prebiotics, synbiotics, and even postbiotics (inanimate microorganisms and/or their components) (defined and reviewed in: [39,40,41]), although the research on postbiotics is thus far scarce. In addition to specific food components or substrates, the dietary composition as a whole, e.g., particular diet patterns, is of interest with regard to potential health effects.

The most evidence of the impact of diet on gut microbiota arises from studies in non-pregnant populations. This is evident for example when the gut microbiota composition of populations consuming distinctive diets are compared, including omnivores, vegetarians, and vegans [42,43]. For example, as *Bifidobacterium* species have different functions, the diet type may result in different physiological significances mediated through microbiota composition and function [43], including the production of short-chain fatty acids (SCFAs), vitamins, and organic acids [44]. This may partly explain the distinctive function of Bifidobacteria in adults and in infants. The strongest evidence thus far points to the association between dietary fiber intake and gut microbiota composition [45]; the finding is supported also by inspection of intervention studies using metagenomics [46], the robust method for sequencing the bacterial genome. The typical finding is an increase in gut microbiota diversity and *Bifidobacterium* due to the consumption of dietary fibers.

Considering energy-yielding nutrients, the focus in research has been on carbohydrates and proteins [47] but the impact of dietary fat on the gut microbiota composition and function is often unrecognized. However, it is important to acknowledge that both experimental and clinical studies indicate a crosstalk between dietary fat and the gut microbiota composition and function [48]. The amount and types of dietary fat may modulate gut microbiota composition (e.g., diversity and abundance of particular bacteria) and its properties (e.g., bacterial adhesion) and metabolites (e.g., SCFAs) produced. This can induce metabolic responses such as a regulation of low-grade systemic inflammation, where both the amount and type of dietary fat are of importance. For example, we demonstrated in a study in pregnant women that the intake of fiber was consistently positive and that the intake of the total fat and different fat types were negatively associated with gut microbiota diversity and richness, with the n-3 LC-PUFAs behaving differently than other types of fats [49]. Interestingly, higher gut microbiota richness and nutrient intakes were linked to a lower level of low-grade inflammation marker GlycA in serum, which offers opportunities for dietary modification during pregnancy with the potential of improving the health of the mother and the child [49]. Overall diet composition or pattern is of importance, as we do not eat individual foods or nutrients, but rather their combinations. Higher scores in the index of diet quality [50,51], which describes an overall healthy diet with reference to dietary recommendations or dietary inflammatory index, which describes dietary inflammatory potential [52], have been shown to associate with higher gut microbiota diversity.

### 4.2. Pregnancy

Diet modification during pregnancy indicates an opportunity to modify the maternal gut microbiota with potential impacts on metabolic, immunological, and clinical outcomes of both the mother and the child. The evidence on diet—gut microbiota relations during pregnancy is thus far scarce. We identified two systematic review articles [53,54] on the topic. The role of a diet high in fat, fat-soluble vitamins, and fiber, as the nutrients that are associated with the gut microbiota composition, were highlighted: high-fiber diets increase microbial diversity, whilst high-fat diets show the opposite effect [54]. Considering carbohydrate intakes, positive associations with Proteobacteria, Bacteroidetes, and Firmicutes were reported, whilst fat intake was associated negatively with Proteobacteria and Bacteroidetes and positively with Firmicutes [53]. Also, some connections between specific bacteria and vitamins were reported [53].

One intervention study with fish oil and/or probiotics demonstrated changes in the relative abundance of bacterial species over the pregnancy, which interestingly were dependent on the maternal gestational diabetes mellitus status [22]. Women without gestational diabetes mellitus manifested changes in the relative abundance of bacterial species over pregnancy, particularly those receiving the combination of fish oil and probiotics. The intervention also exerted effects on many low-abundant vaginal bacteria: Fish oil consumption resulted in a lower abundance of potential pathibionts, like *Ureaplasma urealyticum,* and probiotics to lower the abundance of *Ureaplasma urealyticum* and *Prevotella disiens* [55].

Two notions may be highlighted from these studies. Firstly, gestational diabetes mellitus may disturb maternal gut microbiota flexibility and thus limit the capacity of these women to respond to diet [18,21,22]. This is of importance as the gut microbiota of the children born to women with gestational diabetes mellitus may be altered [56,57]. These results further highlight the evidence for the programming effect of pregnancy events on the child’s gut microbiota and potentially on subsequent health. On the other hand, maternal health conditions, like obesity or gestational diabetes mellitus, may limit the ability to influence the gut microbiota or health by dietary means. Aberrations both in gut microbiota and metabolism are exacerbated in women with obesity compared to those who are overweight [58] or have a normal weight [9]. Similarly, mothers’ weight gain during pregnancy may modify the microbiota [9,20].

The second notion that deserves discussion is the interaction of dietary compounds. One of the first observations here was our finding that maternal dietary composition, namely increased intakes of retinol, calcium, and zinc with perinatal administration of specific strains of probiotics, reduced the risk of atopic eczema, whilst an increase in intake of ascorbic acid increased the likelihood of atopic eczema [59]. The insightful discussion may be found in the original article, but briefly, these effects may arise, e.g., from the regulatory role of the nutrients in innate immunity or from their antioxidative properties. One mechanism for the diet—gut microbiota interaction may result from the function of the pattern recognition receptors. Indeed, pattern recognition receptor soluble(s) CD14 has the main task in binding lipopolysaccharides and thus antimicrobial host defense, but it also binds phospholipids and therefore provides a lipid transfer system with the potential to modify immune events through phospholipid LC-PUFAs. Here, we demonstrated that sCD14 correlated with LC-PUFAs, and again with prostaglandin E2 in the mother’s serum and breast milk, indicating that by engaging in the signaling routes, these may interact in immunomodulation [60].

Taking these observations together, joint effects of nutrients and probiotics need to be considered in clinical trials. This calls for an evaluation of dietary intake in the probiotics intervention studies. Recent demonstrations of such interactions include the finding that combined probiotics and fish oil intervention during pregnancy resulted in more changes than fish oil alone in maternal metabolites as analyzed by omics methods NMR metabolomics, whilst no effects were seen with probiotics alone [61]. The distinct intervention effects were also seen when the gut microbiota [22] or vaginal microbiota [55] were evaluated. Maternal dietary intake and the interactions with other nutrients may be of significance, particularly for infant brain development. One mechanism may be that the placenta selectively transfers LC-PUFAs from the mother to the fetus [62].

Considering clinical findings, probiotics (*Lacticaseibacillus rhamnosus* GG and *Bifidobacterium lactis*) intervention during pregnancy lowered the risk of gestational diabetes mellitus [63] and regulated glucose metabolism of the pregnant women [64]. Since this study, several studies with different probiotics have been conducted, although with controversial findings [65]. Nevertheless, a recent meta-analysis of 11 randomized controlled trials concluded that specific probiotics improve glucose metabolism during pregnancy [66]. The effects of probiotic consumption may extend beyond pregnancy as the intervention lowered the risk of central adiposity at six months postpartum, although the consumption of probiotics, healthy eating patterns, and BMI prior to pregnancy were strong determinants of postpartum BMI as studied at 12 months postpartum [67]. One study indicated the benefits of consuming probiotics during pregnancy (*Lacticaseibacillus rhamnosus* HN001) in lowering depression and anxiety scores in the postpartum period [68].

The benefits of maternal probiotics consumption on child health may be stronger than on maternal health as the developing immune system of the child is malleable. Evidence exists for lowering the risk of overweightness [69] even at the age of 10 years [70] or risk of allergy [71], after perinatal administration of probiotics. With regard to allergic disease, Alemu and co-workers [72] summarized the evidence (including also a range of maternal outcomes) by conducting an umbrella review of systematic reviews and meta-analyses of randomized controlled trials. The authors concluded that probiotic consumption by pregnant and lactating women was beneficial in lowering the risk of atopic dermatitis and eczema. The World Allergy Organization guideline panel suggested the provision of probiotics for women during pregnancy and breastfeeding if their infants had a high risk for developing allergy, as well as to these high-risk infants [73]. Based on the studies, lactobasilli appear to be the most effective probiotics, but no recommendation was given on which probiotics should be used.

### 4.3. Breastfeeding

Breastfeeding is the natural mode of feeding an infant. Of interest here is that breast milk has the capacity to prime the maturation of an infant’s immune system and breast milk may also be considered a mediator of microbial impacts on child gut microbiota and subsequently on health. This may take place via several breast milk components including microbes, HMOs, immune mediators like cytokines, and fats including LC-PUFAs.

A large number of bacterial and archaeal species, 820, in the systematic review by Togo and co-workers, were identified in breast milk, with the majority of these belonging to the phyla Proteobacteria, Firmicutes, Actinobacteria, and Bacteroidetes [74]. The most frequently reported species were *Staphylococcus aureus*, *Staphylococcus epidermidis*, *Streptococcus agalactiae*, *Cutibacterium acnes*, *Enterococcus faecalis*, *Bifidobacterium breve*, *Escherichia coli*, *Streptococcus sanguini*, *Lactobacillus gasseri,* and *Salmonella enterica*. The microbes in breast milk likely originate from the entero-mammary pathway, i.e., the transfer of microbes from the maternal gut to the mammary gland [75]. Another route for breast milk microbiota is the oro-mammary pathway, which is confirmed by the similarity of milk and maternal oral microbiota [76].

It may be possible to modify breast milk microbiota by maternal interventions. A systematic review indicated that the maternal consumption of probiotics increased the detection rate and abundance of beneficial bacteria and decreased those of pathogenic bacteria in the breast milk [77]. Similar conclusions were made by Zaidi and co-workers [78], but they further suggested that the effects of maternal probiotic supplementation are dependent on the health status of the mothers, counting the risk of mastitis, or suffering from mastitis [78]. In one study, prenatal vitamin C supplement was positively associated with milk bacterial diversity and the abundance of *Veillonella* and *Granulicatella* and negatively associated with the abundance of *Finegoldia* [79]. In the same study, fish oil supplement use was positively associated with the abundance of *Streptococcus*.

HMOs are unconjugated glycans, highly abundant in and unique to human milk, and they have prebiotic (bifidogenic) and antiadhesive antimicrobial properties, and they serve as soluble decoy receptors [80]. HMOs may modify the infant gut microbiota [81], lower the risk of infections [82], and impact the child’s development, including neurocognitive development via the gut–brain axis, thereby contributing to the child’s later health. Thus far, the evidence has arisen mainly from animal experimental studies or a few observational studies [83,84]. The extent to which the maternal diet may impact HMOs is poorly known (reviewed in: [85]). A maternal high-fat diet compared to a high-carbohydrate diet may decrease concentrations of sialylated HMOs and supplementation of diet with vitamin A or multivitamins may also modify breast milk HMOs. In one further study, maternal probiotic administration resulted in changes in colostrum HMOs, including an increase in the concentrations of 3-fucosyllactose and 3′-sialyllactose [86].

Breast milk immune mediators include immunoglobulins, cytokines, and chemokines, which are important for the development and maturation of an infant’s immune system [87,88]. Some studies have investigated the impact of probiotics on breast milk immune mediators. These have shown an increase in concentrations of colostrum adiponectin [89] or breast milk transforming growth factor-beta [90]. In our recent study in which we measured 16 immune markers, the intervention effect was seen only in a few markers: maternal consumption of combined fish oil and probiotics resulted in higher concentrations of interleukin(IL)-12p70 than probiotics consumption on its own and higher FMS-like tyrosine kinase 3 ligands than fish oil or probiotics alone, but after multiple corrections, the statistical significances were lost [36]. Also, in another study, only minor effects were seen in breast milk cytokines followed by probiotics consumption during lactation [91].

Interestingly, maternal health conditions may also influence the breast milk composition. Mother’s gestational diabetes mellitus increased the likelihood of the colostrum adiponectin level being lowered [89]. In the study by Soukka and co-workers [36], multivariate linear models were performed, and these revealed significant associations between perinatal use of medication and several colostrum immune mediators. This may be an important point to consider as the use of medication during the perinatal period appears to be common and may interfere with the immune system maturation of the child and thereby subsequent health.

## 5. Summary

**Figure 2:**
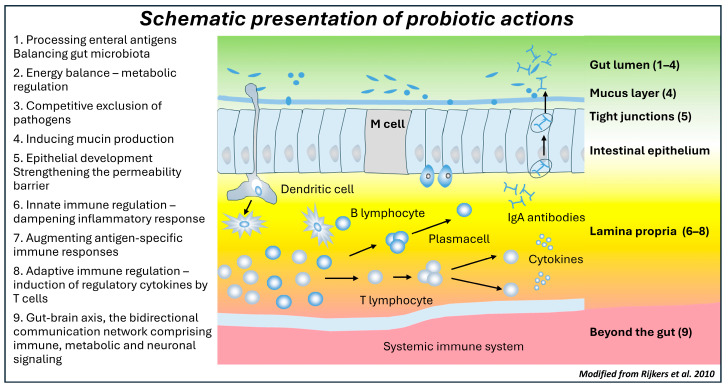
Mechanistic notions on diet-gut microbiota interactions (Modified from [40]). The current evidence from clinical trials demonstrates the action of probiotics in the gut lumen, gut barrier function, and the mucosal immune system (green color), while the evidence of the impact beyond the gut is limited (red color).

Early environments and exposures by the enteral route induce adaptive modifications in the microbiota composition and activity. Microbiota changes, again, control energy acquisition and storage and contribute to the gut barrier function and the host immune defenses (Figure 2). Epidemiological evidence links the risk of NCD inextricably to a shift in the microbiome early in life, by assessing clinical practices known to shift the gut microbiota, such as delivery by cesarean section. However, the proof of causality requires experimental documentation of the mechanisms and clinical intervention studies in populations with a defined risk of NCD. Thus far, we have clinical evidence from probiotic intervention studies in children at risk of allergic disease, the first NCD to manifest itself. Randomized clinical trials emphasize the importance of perinatal intervention, i.e., a continuum from pregnancy to early infancy, and breastfeeding (Figure 1). Similarly, being overweight or obese has a clear intergenerational aspect, and reversing this cycle would necessitate microbiota modulation starting from pregnancy (Figure 1). Parallel data are emerging for a variety of other chronic illnesses including asthma, allergies, and autoimmunity.

While the nutritional microbiota modulation has solid preliminary evidence to support its rationale concerning the impact on the child’s health via the mother’s health and weight gain during pregnancy, as well as the mode of delivery and breastfeeding, with the microbiome as a key mechanism; more data on the clinical benefit of other modes of microbiota modulation are needed. These include maternal microbiota seeding and vaginal and gut microbiota, in view of the distinctions in microbiota composition in adults and infants on the one hand and the gut and vagina on the other. Close skin contact, an important part of neonate/infant care, invites research on microbiota exposures through family members and the home environment, above the diet.

Not only may the nutritional value of the food have an impact on the gut microbiota but also its packaging: experimental studies indicate that microplastics and their co-contaminants may carry an NCD risk. Microplastics can also serve as carriers of antibiotic-resistant bacteria and act to create a portal of entry for the transfer of chemicals and toxins present in or on microparticles [92], identified as a Trojan horse effect on the developing gut microbiota.

Finally, a recent follow-up study documents that the gut microbiota of pregnant women has changed over time [25], in line with the current decline in environmental biodiversity. Indeed, the disappearing microbiota hypothesis rationalizes the emergence of NCDs by pointing to the depletion of microbial contact in the modern industrialized world, resulting from changes in hygiene, antibiotic use, diet, lifestyle, and living conditions, i.e., our surroundings and environment (Figure 1).

## 6. Conclusions and Key Takeaways

Our conception of microbes is changing from the traditional view that a microbe equals a pathogen causing disease to the concept of host–microbe interaction, implying a benefit of the microbial presence to the host physiology and health. The strong association between early nutrition and the compositional development of the gut microbiota, both impacting on the individual’s later health, invites the idea of next-generation personalized diets based on individual risk circumstances. The NCD epidemic, particularly allergy and obesity, represents a fast-moving target and microbiota modulation may offer an innovative solution to halt it from the outset.

## Data Availability

No new data were created or analyzed in this study. Data sharing is not applicable to this article.
